# Compensatory Function of the Subtalar Joint for Lower Extremity Malalignment

**DOI:** 10.1155/2019/7656878

**Published:** 2019-02-24

**Authors:** Kensei Yoshimoto, Masahiko Noguchi, Akifumi Yamada, Yuki Nasu

**Affiliations:** Orthopaedic Foot and Ankle Center, Shiseikai Daini Hospital, 5-19-1 Kamisoshigaya, Setagaya-ku, Tokyo, 157-8550, Japan

## Abstract

It is important to evaluate the subtalar joint and hip-knee-ankle alignment to understand lower extremity alignment. In this review, we focused on the compensatory changes in the subtalar joint alignment for the deformity of the knee and ankle joint, reviewing previous research. The subtalar joint alignment was compensatory valgus in patients with varus knee and ankle deformity, whereas it was uncertain whether the subtalar joint alignment was compensatory varus in patients with valgus knee and ankle deformity. The subtalar joint valgus alignment improved after total knee arthroplasty or high tibial osteotomy for varus knee deformity, even if the deformity was severe. In contrast, whether the subtalar joint alignment changed after the surgery for ankle or valgus knee deformity has not been considered. Further research on the compensatory function of the subtalar joint is needed.

## 1. Introduction

When treatment for lower extremity malalignment is needed, it is important to evaluate the lower extremity alignment correctly. Many studies assessed only the hip-knee-ankle alignment, such as the femorotibial alignment or the mechanical axis running from the center of the femoral head to that of the ankle [1–3]. However, weight-bearing on the lower extremity runs from the hip to the knee, ankle, and foot, and then to the ground. As calcaneus is in contact with the ground, it is necessary to evaluate the alignment of the subtalar joint in addition to the hip-knee-ankle alignment to correctly measure the lower extremity alignment [4].

Each joint of the lower extremity compensates the malalignment caused by deformities of the other joints [10, 11, 20]. In particular, recent studies have discussed the compensatory function of the subtalar joint [7, 13, 19]: several reports show that the subtalar joint compensates for the deformities of the knee and ankle joints [10, 12, 17, 18, 20], and the subtalar joint alignment which was 2°-6° valgus in healthy legs [21, 22] changed after surgery to correct these deformities [7, 14, 16]. When surgery for knee or ankle deformity is needed, it is helpful for surgical planning to understand the mechanism of subtalar joint compensation and how the subtalar joint alignment changes after surgery.

In this literature review, we discuss the compensatory function of the subtalar joint in patients with deformities of the lower extremity, reviewing previous research.

## 2. Radiographic Assessment of the Hindfoot Alignment

**Figure 1:**
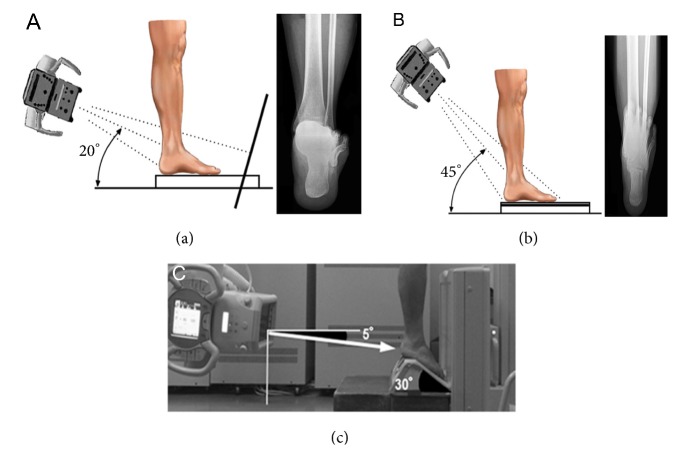
(a) The inclination angle of the beam is 20° to the floor. The film cassette is perpendicular to the central beam of the radiation source. (b) The film cassette is lying on the floor and the subject is standing on the film cassette. The inclination angle of the beam is 45° to the floor. (a) and (b) are reproduced from Reilingh et al. 2010 [5] [under the Creative Commons Attribution License/public domain]. (c) Participants stood on a radiolucent platform with equal weight on both feet. This platform was flat in the rear part and inclined by 30° in the front part, so that the midfoot and forefoot of participants were planter-flexed. The X-ray beam was oriented down 5° from the horizontal position. (c) is reproduced from Ikoma et al. 2013 [6] [under the Creative Commons Attribution License/public domain].

**Figure 2:**
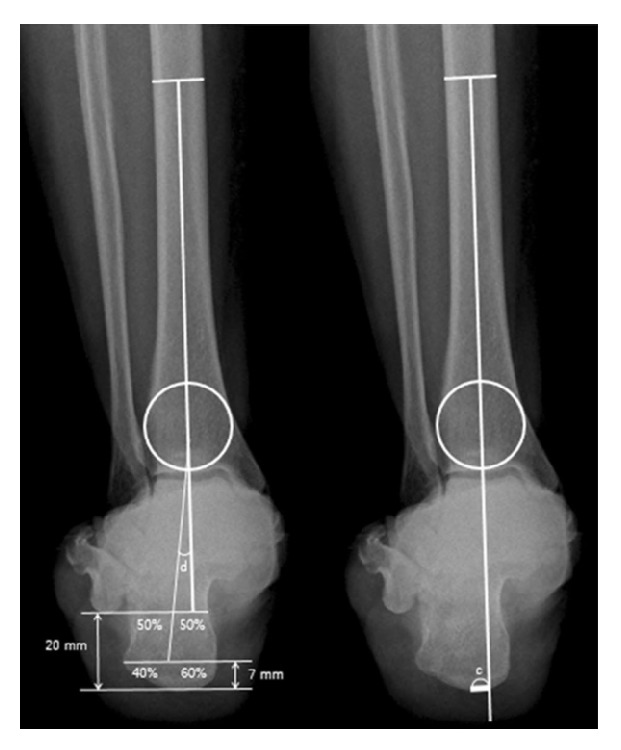
Heel alignment angle (HA) was defined as the angle (d) between the tibial axis and the calcaneal axis. Heel alignment distance (HD) was defined as the distance (c) between the contact point of the heel and the intersection of the extended tibial axis and the distal part of the calcaneus. Figure 2 is reproduced from Jeong et al. 2018 [7].

**Figure 3:**
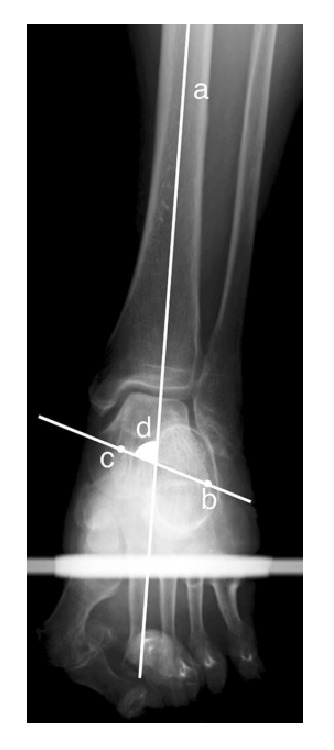
The measurement of the V-V angle. Line a indicates the long axis of the tibia. Point b indicates the lateral extremity of the calcaneus at the posterior surface of the talocalcaneal joint. Point c indicates the superior margin of the sustentaculum tali. Angle d is the V-V angle. Figure 3 is reproduced from Takenaka et al. 2015 [8] [under the Creative Commons Attribution License/public domain].

**Figure 4:**
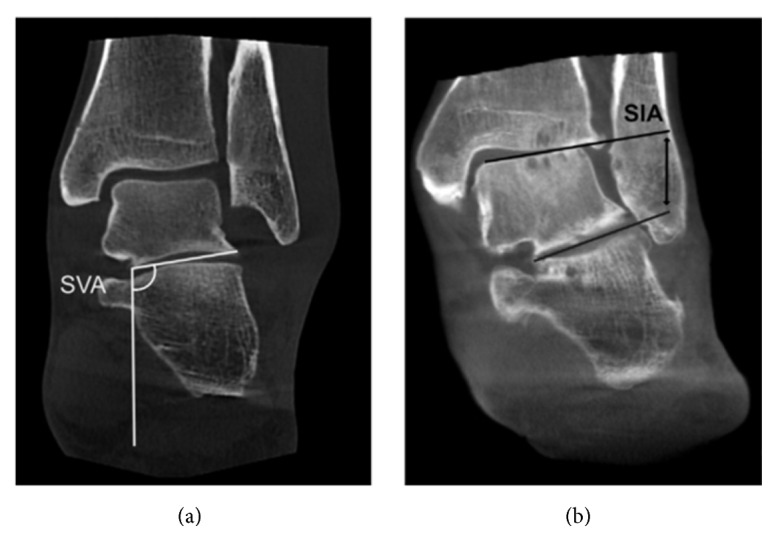
(a) Corresponding weight-bearing computed tomography (CT) scan. The orientation of the subtalar joint to the ground was assessed using the subtalar vertical angle (SVA). (b) Corresponding weight-bearing CT scan. The subtalar inclination angle (SIA) was used to assess the inclination of the subtalar joint. Figure 4 is reproduced from Krahenbuhl et al. 2017 [9].

Several methods can be used for the radiographic imaging of the coronal plane alignment of the subtalar joint or hindfoot. Cobey [23] and Saltzman et al. [24] reported the hindfoot alignment view: subjects stood on a floor, and an X-ray beam with an inclination angle of 20° to the floor was directed from the posterior to the anterior side. A modified method was also reported, with a film cassette lying on the floor and the X-ray beam directed at it with an inclination angle of 45° [5, 25] (Figure 1(b)). In these methods, the hindfoot alignment was usually evaluated using the heel alignment distance (HD) as the distance between the contact point of the heel and the intersection of the extended tibial axis and the distal part of the calcaneus [7, 19, 24], and the heel alignment angle (HA) measured as the angle between the axis of the distal tibia and the axis of the calcaneus [11, 12, 15, 26, 27] (Figure 2). HA is also referred to as hindfoot alignment angle or tibiocalcaneal angle. On the other hand, Ikoma et al. [6] reported a different method, whereby subjects stood on a radiolucent platform, which was flat in the rear part and inclined by 30° in the front part. The X-ray beam was oriented down 5° from the horizontal position (Figure 1(c)). This method assessed the hindfoot alignment using the V-V angle, measured as the angle between the axis of the line from the top of the sustentaculum tali to the lateral-inferior end of the posterior facet of the calcaneus and tibial axis (Figure 3). Moreover, the utility of weight-bearing computed tomography (CT) scans has also been recently suggested [28, 29]. In CT scans, the subtalar vertical angle (SVA) and subtalar inclination angle (SIA) were measured for evaluating the subtalar joint alignment (Figure 4).

## 3. Compensatory Function of the Subtalar Joint for Deformity of the Knee

Norton et al. [12] showed that there was a significant correlation between knee and hindfoot alignment, and a moderately strong correlation in patients with knee deformity, and this correlation was remarkable with larger knee deformity (≥ 10°). The correlation coefficient between hindfoot angle and mechanical axis angle was -0.413 and was -0.536 for those with larger knee deformity. These results indicate that the hindfoot alignment was compensatory valgus in patients with varus knee osteoarthritis (OA) and was varus in patients with valgus knee OA. Several reports supported this hindfoot compensatory function in patients with varus knee OA [11, 14]. However, hindfoot compensation for valgus knee OA could not be found in the report by Mullaji et al. [11].

Nakada et al. [15] demonstrated the subtalar joint compensation for the deformity of knee joints with rheumatoid arthritis (RA). The positive correlation between the femorotibial angle (FTA) and the HA only existed in knees with a Larsen grade≥ 4 (r= 0.544), but not in knees with a Larsen grade≤ 3 (r= 0.180). In measurement of the FTA, a lesser value is considered as valgus knee and bigger as varus. In measurement of the HA, a lesser value is considered as varus hindfoot and bigger as valgus. This correlation was stronger in patients with less damaged subtalar joints (Larsen grade≤ 3) (r= 0.705).

## 4. Compensatory Function of the Subtalar Joint for Deformity of the Ankle

Several reports showed that the subtalar joint could compensate the varus ankle OA [9, 17–19]. The subtalar inclination angle (SIA) increased from Takakura Stages 1 to 3a [30] (2.9° ± 7.0° in Stage 1, 8.0° ± 8.6° in Stage 2, and 11.5° ± 5.7° in Stage 3a) compared to the control (1.5° ± 5.9°) and decreased at Stages 3b and 4 (4.0° ± 9.2° in Stage 3b and 3.0° ± 9.8° in Stage 4) [17]. This suggested that the subtalar joint was compensatory valgus in patients with early to intermediate Takakura stage but could not compensate in patients with the end stage. In contrast, Krahenbuhl et al. [9] found that the subtalar joint compensation for the varus ankle OA was independent of the stage of ankle joint osteoarthritis, extent of the talar tilt in the ankle joint mortise.

In patients with valgus ankle OA, the subtalar joint compensation might be small [9, 19]. Wang et al. [19] showed that only 38.6% of valgus ankles were compensated by subtalar joints (HA < -7.7°), whereas 53% of varus ankles were compensated (HA > -13.1°). Krahenbuhl et al. [9] could not find the subtalar joint compensation in patients with valgus ankle OA. In their study using weight-bearing CT scans, SVA and SIA measurement revealed that the subtalar joint was valgus synchronizing the valgus ankle deformity.

## 5. The Subtalar Joint Alignment after Surgery for the Knee Malalignment

Chandler et al. [10] first described that the hindfoot varus or valgus angle changed after the correction of knee deformity with total knee arthroplasty (TKA), showing that the hindfoot compensated the knee deformity. However, they did not clearly explain the correlation between them. Thereafter, several reports found that the compensatory subtalar valgus was corrected after TKA in patients with knee OA [7, 8, 11, 13, 14, 31]. Hara et al. [31] and Takenaka et al. [8] evaluated the subtalar joint alignment using the V-V angle, which averaged a normal value of 76.0° [6] and showed significant improvement in the subtalar joint alignment in patients with subtalar valgus (V-V angle ≥ 76.0°) from 80.5° ± 3.1° to 78.6° ± 3.7° three weeks after TKA and further improved to 77.1° ± 2.7° one year after TKA. However, in patients with subtalar varus (V-V angle < 76.0°), the preoperative V-V angle (72.7° ± 2.6°) did not improve three weeks and one year after TKA (72.3° ± 3.3°, and 73.5° ± 3.0°, respectively). In another report from Cho et al. [14], greater improvements of hindfoot valgus were found in patients with a severe varus deformity of the knee joint (varus ≥ 10°). Greater improvement of the mechanical axis angle from 13.9° ± 3.7° varus to 2.6° ± 3.5° varus in these patients might result in a greater improvement of hindfoot valgus from 6.5° ± 3.8° valgus to 2.5° ± 4.1° valgus. Similar results were observed by Jeong et al. [7], who found a correlation between the pre-postoperative variance in the mechanical axis angle and the ground-talar dome angle, HA and HD (r = 0.7, -0.348 and -0.418, respectively). Okamoto et al. [13] focused on the American Orthopaedic Foot and Ankle Society (AOFAS) ankle–hindfoot scale in addition to the hindfoot alignment after TKA. They also evaluated calcaneal pitch and naviculocuboid overlap in plane weight-bearing lateral foot radiograph as the index of hindfoot alignment. They found that calcaneal pitch, naviculocuboid overlap, and AOFAS ankle–hindfoot scale with moderate varus deformity of the knee (≤ 6° varus) improved after TKA, but those with severe varus deformity of the knee (> 6°) did not improve after TKA.

In contrast, only one report assessed the subtalar joint alignment after TKA in patients with valgus knee OA [11]. They showed that the subtalar joint was valgus before TKA and remained valgus after TKA. However, because their study included quite a small number (n= 12) of patients with valgus OA, further studies with larger sample sizes are needed.

The change of the subtalar joint alignment after tibial osteotomy for knee malalignment was also assessed. Choi et al. [16] evaluated the hindfoot alignment after high tibial osteotomy. They found that the preoperative degree of hindfoot valgus deviation (7.8° valgus) decreased progressively 3 months, 6 months, and 12 months after HTO (4.0°, 3.4°, and 2.3° valgus, respectively).

## 6. The Subtalar Joint Alignment after Surgery for the Ankle Malalignment

There is only one report by Choi et al. [16] which evaluated the hindfoot alignment after surgery for ankle OA. They evaluated the hindfoot alignment after low tibial osteotomy (LTO) [32] for ankle OA. They included patients with severe ankle OA averaged Takakura stage of 3.2 [30], and preoperative mild hindfoot varus deviation (1.0° varus) was seen. After LTO, the hindfoot alignment was changed to valgus deviation without any compensatory mechanism 3 months, 6 months, and 12 months after LTO (4.8°, 4.7°, and 4.8° valgus, respectively).

## 7. Discussion

The subtalar joint alignment was compensatory valgus for varus knee deformity, but this compensatory function remains controversial in the valgus knee deformity (Table 1) [11, 12, 15]. On the other hand, the subtalar joint could compensate for early stage varus ankle OA, but not for end-stage varus ankle OA and valgus ankle OA [9, 17–19]. If the subtalar joint was destroyed, the subtalar joint compensation for deformity in the knee and ankle joints might not occur [15, 19]. Lee et al. [18] reported that patients with a well-preserved subtalar range of motion may better compensate varus ankle OA. These results may indicate that the destroyed subtalar joint was rigid and did not have enough joint movement to compensate for the deformity of the knee and ankle. Nakada et al. [15] assessed the subtalar compensatory alignment for the knee deformity in patients with RA. They found that the subtalar joint could compensate the knee deformity, and this compensation was stronger in patients with less damaged subtalar joints (Larsen grade≤ 3). The subtalar joint without severe destruction could compensate both the varus and valgus deformity of the knee in patients with RA. However, the destructed subtalar joint could not compensate the knee deformity and became varus or valgus synchronizing to the varus or valgus knee deformity.

Surgical procedures such as TKA or HTO for the varus knee OA could also improve the subtalar compensatory malalignment (Table 1) [7, 8, 11, 13, 14, 16]. In particular, greater improvements of hindfoot valgus occurred in patients with a severe varus deformity of the knee joint [14], and the variation of correction of varus knee alignment was significantly correlated with that of valgus subtalar alignment after TKA [7]. In contrast, Okamoto et al. [13] found that subtalar valgus did not improve in severe knee OA. However, they evaluated the subtalar alignment using calcaneal pitch and naviculocuboid overlap in a plane weight-bearing lateral foot radiograph. It was unclear whether these sagittal alignment parameters directly reflected the coronal subtalar joint varus or valgus alignment. Takenaka et al. [8] found that the subtalar joint alignment did not improve after TKA in the subtalar joint varus group. The preoperative subtalar joint varus could indicate that the subtalar joint lost the ability to compensate and result in rigid varus. Therefore, the subtalar joint remained varus after TKA. Although correction of varus knee OA could improve the subtalar valgus alignment, it was unclear whether correction of valgus knee OA could also influence the subtalar joint alignment. Further study is desirable to pursue these additional research gaps.

The influence of surgery for the ankle deformity to the subtalar joint was only evaluated in one study (Table 1) [16] and was quite unclear. They showed that the hindfoot alignment was varus in patients with advanced ankle OA and changed to valgus after LTO. Because the ability of subtalar joint compensation might be lost in advanced ankle OA [17, 18], hindfoot alignment became valgus after LTO, synchronizing to the correction of ankle alignment. Further assessments for change of the subtalar joint alignment at several stages of ankle OA after LTO, total ankle arthroplasty, and ankle arthrodesis could reveal the compensation mechanism of the subtalar joint.

## Figures and Tables

**Table 1:** Review of studies which assessed the subtalar joint compensation for the knee or the ankle deformity.

	Total number	Reasons for deformation	Surgical procedure	Evaluation of subtalar alignment	Findings
Chandler et al. [10]	86	Varus and valgus knee OA	TKA	plane radiographs	The subtalar joint compensated the deformity of the knee. There was no correlation between the knee and subtalar joint alignment.

Mullaji et al. [11]	165	Varus and valgus knee OA	TKA	plane radiographs	The hindfoot valgus decreased after TKA. 87% of the subtalar joint continued to have valgus alignment even after TKA.

Norton et al. [12]	401	Varus and valgus knee OA	-	plane radiographs	Most of the compensation to angular deformity at the knee occurred in the subtalar joint. There was a correlation between knee and hindfoot deformities in patients with a larger knee deformity (≥ 10°).

Takenaka et al. [8]	71	Varus knee OA	TKA	plane radiographs	The subtalar joint alignment improved 3 weeks after TKA in the subtalar joint valgus group, and further improvement was noted 1 year following TKA. The subtalar joint alignment did not improve after TKA in the subtalar joint varus group.

Okamoto et al. [13]	75	Varus knee OA	TKA	plane radiographs	Calcaneal pitch, naviculocuboid overlap and hindfoot pain with moderate varus deformity of knee (≤ 6° varus) improved after TKA, but those with severe varus deformity of knee (> 6°) did not improve after TKA.

Cho et al. [14]	195	Varus knee OA	TKA	plane radiographs	Greater improvements of hindfoot valgus occurred in patients with a severe varus deformity of the knee joint (≥ 10°) after TKA. There was no further improvement in hindfoot alignment between 6 weeks and 2 years post-operatively.

Jeong et al. [7]	375	Varus knee OA	TKA	plane radiographs	The variation of correction of varus knee alignment was significantly correlated with that of the valgus ankle and subtalar alignment after TKA.

Nakada et al. [15]	205	Knee RA with varus and valgus deformity	-	plane radiographs	Correlation exists between the knee and hindfoot alignments only in knees with a Larsen grade ≥ 4. This correlation was stronger in patients with less damaged subtalar joints (Larsen grade ≤ 3).

Choi et al. [16]	38 for HTO 46 for LTO	Varus knee and ankle OA	HTO and LTO	plane radiographs	Hindfoot alignment was valgus in patients with advanced arthritis of the knee, but mild hindfoot varus deviation was seen in patients with advanced arthritis of the ankle. Preoperative hindfoot valgus deviation was decreased after HTO, whereas the hindfoot alignment was changed to valgus deviation synchronizing to the ankle alignment after LTO.

Hayashi et al. [17]	133	Varus ankle OA	-	plane radiographs	The subtalar joint compensates varus deformity of the ankle at the intermediate stage ankle OA, but not at the end stage.

Lee et al. [18]	154	Varus ankle OA	-	plane radiographs	Hindfoot alignment was valgus in many ankles in stage II and IIIA, and the mean value was varus in stage IIIB and IV.

Wang et al. [19]	233	Varus and valgus ankle OA	-	plane radiographs	Compensation of the subtalar joint was identified in 39% of valgus ankle OA and 53% of varus ankle OA. The patients with no or mild degenerative changes of the subtalar joint may compensate the ankle malalignment

Krahenbuhl et al. [9]	88	Varus and valgus ankle OA	-	computed tomography	Compensation of the subtalar joint for the ankle deformity could only be verified for varus ankle osteoarthritis and not for valgus ankle osteoarthritis. Subtalar joint alignment had no influence on the stage of ankle osteoarthritis, extent of the tibiotalar tilt and stage of subtalar joint osteoarthritis.

OA: osteoarthritis, TKA: total knee arthroplasty, HTO: high tibial osteotomy, LTO: low tibial osteotomy
